# Two New Three-Dimensional Pillared-Layer Co(II) and Cu(II) Frameworks Involving a [M_2_(EO-N_3_)_2_] Motif from a Semi-Flexible *N*-Donor Ligand, 5,5′-Bipyrimidin: Syntheses, Structures and Magnetic Properties

**DOI:** 10.3390/polym10030229

**Published:** 2018-02-26

**Authors:** Zu-Zhen Zhang, Han-Ting Chang, Yi Lin Kuo, Gene-Hsiang Lee, Chen-I Yang

**Affiliations:** 1 Department of Chemistry, Tunghai University, Taichung 407, Taiwan; a7902112@gmail.com (Z.-Z.Z.); tamy1356789@gmail.com (H.-T.C.); rj810905@gmail.com (Y.L.K.); 2 Instrumentation Center, National Taiwan University, Taipei 106, Taiwan; ghlee@ntu.edu.tw

**Keywords:** semi-flexible *N*-donor ligand, coordination polymer, magnetic properties, spin canting, metamagnetism, spin-flop

## Abstract

Two new three-dimensional (3D) Co(II)- and Cu(II)-azido frameworks, [Co_2_(N_3_)_4_(bpym)_2_]*_n_* (**1**) and [Cu_2_(N_3_)_4_(bpym)]*_n_* (**2**), were successfully synthesized by introducing a semi-flexible *N*-donor ligand, 5,5′-bipyrimidin (bpym), with different bridging modes and orientations. Compounds **1** and **2** were structurally characterized by X-ray crystallography, IR spectroscopy, thermogravimetry and elemental analysis. Compounds **1** and **2** are 3D pillared-layer frameworks with double end-on (EO) azido bridged dinuclear motifs, [M_2_(EO-N_3_)_2_]. In Compound **1**, the bpym ligands show *trans* *μ*_2_-bridging mode and the role as pillars to connect the Co(II)-azido layers, composed of [Co_2_(EO-N_3_)_2_] motifs and single end-to-end (EE) azido bridges, to a 3D network with **BN** topology. In contrast, in **2**, the bpym ligand adopts a twisted *μ*_4_-bridging mode, which not only connects the adjacent [Cu_2_(EO-N_3_)_2_] units to a layer, but also functions as a pillar for the layers of the 3D structure. The structural diversities between the two types of architectures can be attributed to the coordination geometry preference of the metal ions (octahedral for Co^2+^ and square pyramidal for Cu^2+^). Magnetic investigations revealed that Compound **1** exhibits ferromagnetic-like magnetic ordering due to spin canting with a critical temperature, *T*_C_ = 33.0 K, and furthers the field-induced magnetic transitions of metamagnetism at temperatures below *T*_C_. Compound **2** shows an antiferromagnetic ordering with *T*_N_ = 3.05 K and a field-induced magnetic transition of spin-flop at temperatures below the *T*_N_.

## 1. Introduction

The constructions of functional one-, two- and three-dimensional coordination polymeric materials (CPs) using paramagnetic metal ions are of great interest because of the potential applications in a variety of areas due to their intriguing network topologies [1,2,3,4,5,6,7,8], such as electronic properties, magnetic properties, host-guest chemical properties, ion exchanging behaviors, catalysis, nanotechnology, fluorescence properties, nonlinear optical properties, etc. The abilities for magnetic CPs to establish relationships for structure-property, such as magneto-structural correlations toward molecule-based magnets, are key issues to develop this rapidly growing field of chemistry [9,10,11,12]. In general, the magnetic CPs are synthesized via a bottom-up approach using paramagnetic metal ions and/or metal clusters as building block linked by suitable bridging ligands, which can efficiently transmit magnetic couplings between each metal ion. The short bridging ligands, such as cyanide, carboxylate and azide, as efficient magnetic transmitting ligands, are dominant in the literature [13,14,15,16,17,18]. Thus, enormous efforts on magnetic CPs have been focused on the design of suitable organic ligands and the coordination tendencies of metal centers for the building of diversified extended networks with interesting magnetic properties. The *N*-heterocyclic ligands, such as triazole and tetrazole, are also receiving considerable attention for the preparation of new magnetic CPs [19,20]. The bipyrimidine-based ligand, 2,2′-bipyridine, has been utilized in the construction of magnetic CPs with various architectures, in which the pyrimidyl group not only exhibits the bridging ligands to multiple metal centers in the resulting polymeric structures, but also mediates significant magnetic couplings that may have an unusual magnetic behavior, such as ferromagnetism and spin-canting [21,22,23]. However, CP’s that are prepared from 2,2′-bipyridime usually show low-dimensionality because of a lack of flexibility due to the presence of strong chelating effects.

Thus, in our synthetic approach, we used a semi-flexible bipyrimidyl ligand, 5,5′-bipyridine (bpym), as a bridging ligand. This ligand shows several stimulating characteristics when coordinated to metal ions: (i) the ligand is capable of bridging the group for multiple metal centers to polymeric structures due to its multiple *N*-coordination sites; (ii) the multiform structural configurations of the ligands in nature; the dihedral angles of two pyrimidyl groups and out of the plane of the aromatic ring with coordination metal ions have sufficient flexibility and adaptability to achieve the requirements needed for raising coordination frameworks with varied frameworks, and the multiple *N*-donor backbones may support supramolecular network formation via H-bonding and π–π aromatic interactions; (iii) metal ions bridged by a pyrimidyl group may not only adopt a shorter metal-metal distance, but also exhibit significant magnetic interactions.

Herein, we report on the synthesis and characterization of two new coordination polymers, [Co_2_(N_3_)_4_(bpym)_2_]*_n_* (**1**) and [Cu_2_(N_3_)_4_(bpym)_2_]*_n_* (**2**), prepared using bpym and azide as co-ligands. Both **1** and **2** are made up of 3D frameworks involving a double end-on (EO) azido bridged dinuclear motif, [M_2_(EO-N_3_)_2_], with pillared-layer architectures. The structure of Compound **1**, which is composed of Co(II)-azido layers is constructed from [Co_2_(EO-N_3_)_2_] motifs and single end-to-end (EE) azido bridges and linear *μ*_2_-bpym pillars. In Compound **2**, the bpym ligand adopts a twisted *μ*_4_-bridging mode, which not only connects the adjacent [Cu_2_(EO-N_3_)_2_] units to a layer, but also functions as a pillar between the layers, thus forming a 3D structure. Magnetic investigations revealed that Compound **1** exhibits ferromagnetic-like magnetic ordering attributed to spin canting with a critical temperature of *T*_C_ = 33.0 K and a field-induced metamagnetic transitions below its *T*_C_. Compound **2** shows an antiferromagnetic ordering with *T*_N_ = 3.05 K and a field-induced spin-flop magnetic transition below its *T*_N_.

## 2. Experimental

### 2.1. Materials and Methods 

All reactions were achieved under aerobic situations. Azido compounds are potentially explosive and should be prepared and used only in small amounts and treated with the highest care at all times. The 5,5′-bipyrimidine was synthesized following a procedure reported in [24].

### 2.2. Synthesis of [Co_2_(N_3_)_4_(bpym)_2_]_n_ *(**1**)*

A solution of NaN_3_ (66.9 mg, 1.02 mmol) in water (3 mL) was mixed with a water solution (5 mL) containing Co(NO_3_)_2_·6H_2_O (73.8 mg, 0.25 mmol) and bpym (10.0 mg, 0.06 mmol). A clear solution was obtained after stirring for 5 min. The purple crystals of **1** suitable for X-ray analysis were obtained from the resulting solution after standing at room temperature for a week. The crystals of products were collected by suction filtration, washed with water and dried in air. Yield: 46% (based on bpym). A pattern of the bulk sample obtained by powder X-ray diffraction compared well with the simulation pattern by the single-crystal data (vide infra). Elemental analysis calcd. (%) for C_8_H_6_CoN_10_ (**1**): C, 31.91; H, 2.01; N, 46.51; found: C, 31.60; H, 2.00; N, 46.32. IR data (KBr disk, cm^−1^): 3443(w), 3080(w), 2108(vs), 2075(vs), 2050(vs), 1585(s), 1563(s), 1455(w), 1410(vs), 1360(vs), 1340(s), 1288(s), 1182(s), 1198(s), 1140(w), 1059(w), 1016(s), 908(s), 717(vs), 687(w), 655(vs), 591(w).

### 2.3. Synthesis of [Cu_2_(N_3_)_4_(bpym)]_n_ *(**2**)*

A solution of Cu(NO_3_)_2_·3H_2_O (33 mg, 0.13 mmol) in methanol (5 mL) was carefully layered on top a solution of bpym (10 mg, 0.06 mmol), and NaN_3_ (17 mg, 0.26 mmol) in water (5 mL). It was then allowed to stand for two weeks at room temperature, whereupon block needle crystals of **2** were formed. The crystals of the products were collected by suction filtration, washed with water and dried in air. Yield: 74% (based on bpym). A pattern of the bulk sample obtained by powder X-ray diffraction compared well with the simulation pattern by the single-crystal data (vide infra). Elemental analysis calcd. (%) for C_8_H_6_Cu_2_N_16_ (**2**): C, 21.19; H, 1.33; N, 49.43. Found: C, 21.04; H, 1.29; N, 49.40. IR data (KBr disk, cm^−1^): 3446(w), 3064(w), 3055(w), 3033(w), 2072(vs), 2029(vs), 1590(w), 1566(w), 1455(w), 1408(vs), 1327(w), 1281(vs), 1182(s), 1144(w), 1058(w), 1026(s), 930(w), 921(w), 717(vs), 690(s), 663(vs), 588(w). 

### 2.4. X-ray Crystallography

Diffraction measurements of Compounds **1** and **2** were carried out using a Bruker–Nonius Kappa CCD diffractometer (Bruker, Karlsruhe, Germany) and a Bruker SMART APEX CCD diffractometer (Bruker, Karlsruhe, Germany), respectively, with graphite-monochromated Mo Kα radiation (λ = 0.7107 Å). Absorption corrections were applied by program SADABS. The structures were solved using direct methods and refined against F^2^ by the full-matrix least-squares technique, using the SHELXTL-97 program [25]. All non-hydrogen atoms were refined anisotropically, whereas the hydrogen atoms were fixed in ideal positions and refined isotropically with a riding model. In Compound **1**, the two pyrimidyl rings are treated as in disorder, and the occupancies for atoms of C1, N2, C3, C4, N4, C6 and atoms of C1′, N2′, C3′, C4′, N4′, C6′ are refined as 0.489 and 0.511, respectively. Detail experimental X-ray data collection and the refinements of Compounds **1** and **2** are shown in Table 1, and selected interatomic distances are listed in Table 2.

### 2.5. Physical Measurements

Dc magnetic susceptibility measurements operating at a variable temperature and field were collected on powdered samples on a Quantum Design MPMS-7 SQUID magnetometer (Quantum Design, San Diego, CA, USA) equipped with a 7.0 T magnet and a PPMS magnetometer equipped with a 9.0 T magnet. Diamagnetic corrections were assessed from Pascal’s constants [26] and deducted from the experimental susceptibility data to get the molar paramagnetic susceptibility for both compounds. Elemental analyses of both compounds were collected using an Elemental vario EL III analyzer (Elementar, Langenselbold, Germany). A Seiko Instrument, Inc., EXSTAR 6200 TG/DTA analyzer (Seiko Instruments, Chiba shi, Japan) was used for collecting thermogravimetric (TG) analysis data with a heating rate of 5 °C/min under a nitrogen atmosphere. The powder diffraction measurements of both compounds were recorded on a Siemens D-5000 diffractometer (Siemens, München, Germany) by step mode with a fixed time of 10 s and a step size of 0.02° in θ for Cu-Kα (λ = 1.5406 Å). Fourier transform infrared (FTIR) spectra of both compounds were measured in KBr pellet using a Perkin Elmer Spectrum RX-1 FT-IR Spectrometer (Perkin Elmer, Waltham, USA). 

## 3. Results and Discussion

### 3.1. Syntheses and Characterization of Compounds ***1*** and ***2***

Compounds **1** and **2** were both prepared by the reacting M(II) nitride, sodium azide and the bpym ligand in H_2_O or MeOH/H_2_O mixed solutions at room temperature. Compound **1** was obtained by using a starting Co:azide:bpym molar ratio of 1:4:0.5, and Compound **2** was synthesized by using a starting Cu:azide:bpym molar ratio of 1:2:0.5. In addition to a solvent effect in crystallization, the use of a larger azido molar ratio enhanced the formation of Co-N_3_ coordination bonds, and the resulting (EE-N_3_)_2_[Co_2_(EO-N_3_)] layers could be further crosslinked through the *trans μ*-*N*,*N*′-bpym bridge. In contrast, the use of a lower azide molar ratio may not favor the same layer structure. In this case, the [Cu_2_(EO-N_3_)] units are connected to each other through the *μ*-pym ring bridge to form a [Cu_2_(EO-N_3_)]-(pym)_2_ layer, in which the bpym ligands not only bridge the [Cu_2_(EO-N_3_)] units to a layered structure, but also connect adjacent layers to form a 3D framework with a *μ*_4_-*N*,*N*′,*N*′′,*N*′′′-bridging mode. The phase purity of the bulk samples of both compounds was independently confirmed by the data of elemental analysis and PXRD (Figure S1). The thermal stability of Compounds **1** and **2** was also characterized by TG analysis (Figure S2), in which the TG curves of **1** and **2** show large weight loss at about 220 °C and 180 °C, respectively, indicating the decomposition of frameworks. On the basis of single-crystal X-ray diffraction data and elemental analysis results, the formulas for the compounds were determined to be [M_2_(N_3_)_4_(bpym)]*_n_* (M = Co and Cu for Compounds **1** and **2**, respectively). Both compounds show a very strong band around ν = 2075 cm^−1^, which was assigned to a ν_as_(N_3_) vibration. The medium IR band around ν = 3050 cm^−1^ is a ν(C–H) vibration characteristic of the bpym ligand.

### 3.2. Description of Structure

#### 3.2.1. Crystal Structures of Compound **1**

**Figure 1:**
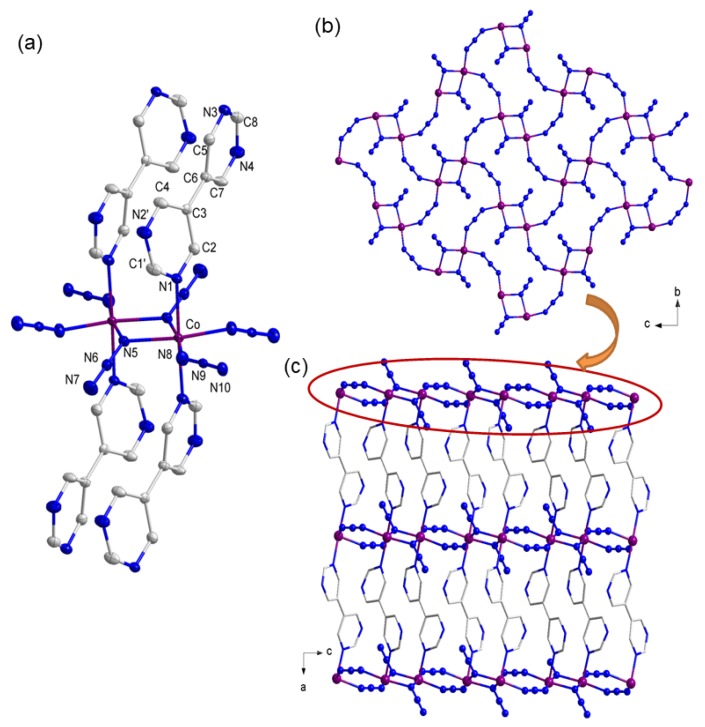
(**a**) The local coordination environment of Compound **1**; (**b**) the 2D Co_2_-EE-N_3_ layer; (**c**) the pillared-layer 3D structure of **1**. The H atoms of bpym ligand have been omitted for clarity.

**Scheme 1:**
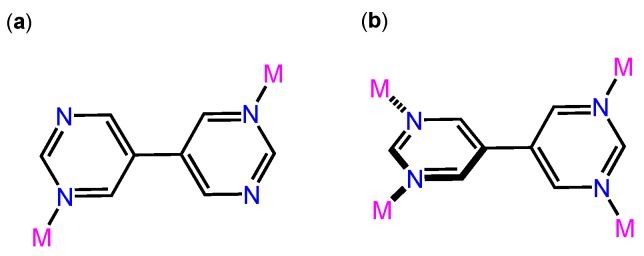
(**a**) The linear *trans*-*μ*_2_-*N*,*N*′-bpym in Compound **1**; (**b**) the twisted *μ*_4_-*N*,*N*′,*N*′′,*N*′′′-bpym in Compound **2**.

**Figure 2:**
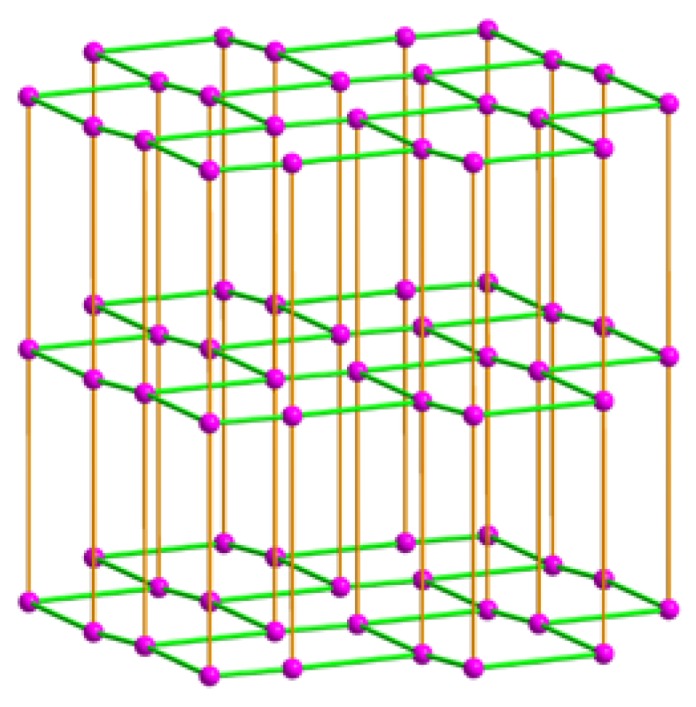
Schematic representation of the five-connected **BN** topologic network of Compound **1**; the bpym are represented by yellow; end-on- (EO) and end-to-end- (EE) azides are represented by green; and Co are represented by purple spheres.

Single crystal X-ray analysis data showed that Compound **1** crystallizes in the *P*2_1_/c space group. The asymmetric unit of **1** contains one crystallographically independent Co(II) ion, one bpym ligand and two azido anions. As depicted in Figure 1a, the Co(II) ion shows a distorted octahedral coordination geometry, where the equatorial positions are occupied by four nitrogen atoms (N5, N5A, N8 and N10) derived from four azido anions and two nitrogen atoms (N5) from an azido anion, and the axial positions are occupied by two nitrogen (N1 and N3) atoms derived from two bpym ligands with Co–N distances in the range 2.113–2.163 Å. Two neighboring Co(II) ions, related by an inversion center, are doubly linked by two EO-N_3_ bridges (N5 and N5A) resulting in a dinuclear [Co_2_(EO-N_3_)_2_] unit, in which the Co–N–Co bridging angle and Co···Co distance are 101.26° and 3.329 Å, respectively. These are the typical values for double EO-N_3_ bridges [27]. Each Co_2_ unit is further connected to four neighboring identical Co_2_ units through four EE-N_3_ anions and results in a 6^3^-**hcb** layer paralleling to the *bc* crystal plane (Figure 1b). The bridging Co–N–N angle in the layer is around 139.3°, with a unique torsion angle of Co–NNN–Co of 5.47°. The metal coordination geometry of each Co_2_ moiety in the layer shows two alternating orientations, with the dihedral angle of the equatorial planes in 35.1° and the Co···Co distance between two EE-N_3_-bridged Co(II) ions in 5.647 Å. Similarly, the dihedral angle of the Co_2_N_2_ plane between each Co_2_ moiety is 34.7°. Due to the alternating orientation of the metal spheres and the non-coplanar bridging of the EE-N_3_ ligand, the Co(II)-N_3_ layer has an undulating shape. As displayed in Figure 1c, the Co(II)-N_3_ layers are connected in a cavity-above-cavity (···AAAA···) fashion into a 3D pillared-layer framework by the bpym ligands, in which the bpym pillar ligands function to link Co(II) ions from neighboring layers in a *trans μ*-*N*,*N′*-bpym mode (Scheme 1a) with a slightly twisted conformation (the dihedral angle of two pyrimidyl rings is 19.5°) resulting in the shortest interlayer Co···Co distance of 9.913 Å. The bpym ligands in **1** are stacked in an overlapping manner to form a 1D infinite array along the *b* crystallographic direction, with weak π–π interactions between pyrimidyl groups. The dihedral angles between the interacting pyrimidyl rings are 5.3 ° and 6.83°, and the separations between the rings are 3.532 Å, and 3.469 Å for centroid-centroid distances. From a topology view, the 3D structure of **1** arises from the stacking of 2D (6,3) layers, where the Co(II) center can be regarded as a five-connected node with three different sets of linkers (double EO-N_3_ bridges, single EE-N_3_ bridges and *μ*-bpym bridges). This connectivity repeats infinitely giving the 3D network of Compound **1** as shown in Figure 2. An analysis using the TOPOS software package [28] indicated that the net of **1** can be rationalized as a uninodal five-connected **BN** net with the Schläfli symbol (4^6^.6^4^) as illustrated in Figure 2.

#### 3.2.2. Crystal Structures of Compound **2**

**Figure 3:**
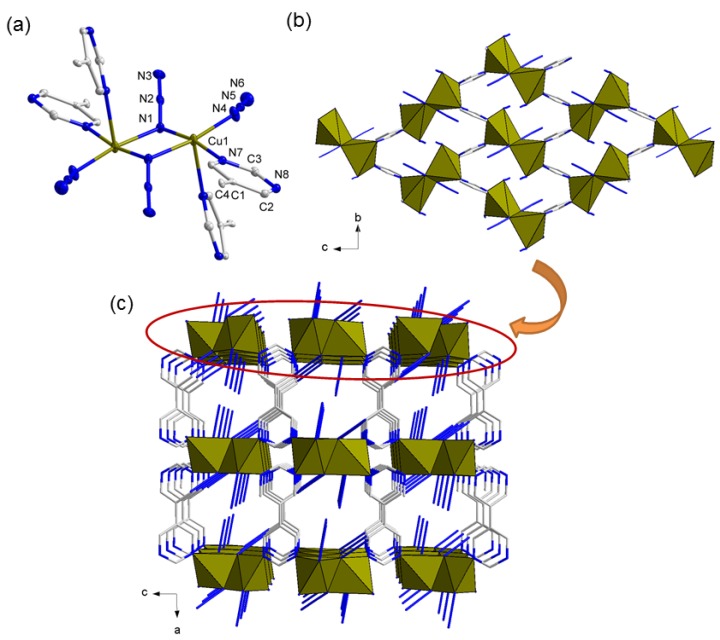
(**a**) The local coordination environment of Compound **2**; (**b**) the 2D Cu_2_-pym layer; (**c**) the pillared-layer 3D structure of **1**. The H atoms of the bpym ligand have been omitted for clarity.

The single crystal X-ray diffraction data revealed that Compound **2** crystallizes in the monoclinic with *C*2/*c* space group. The asymmetric unit of Compound **2** has one crystallographically independent Cu(II) ion, one bpym ligand and two azido anions. As depicted in Figure 3a, the Cu(II) ion is CuN_5_ penta-coordinated with a square pyramidal geometry (τ = 0.01), in which the basal plane is coordinated by N1 and N1A of two EO-N_3_ bridges, by N4 of a terminal bonded azido ligand and by N7 of a bpym ligand, while the apical coordination site is occupied by N8 of a bpym ligand. The Cu–N bond distances of basal positions are in the range of 1.966(2)–2.042(2) Å, which are shorter than in the apical position of 2.528(2) Å, due to the Jahn–Teller (JT) distortion. In the basal plane, the two *trans* N–Cu–N angles are 170.56(8)° and 169.75(7)°, while the *cis* N–Cu–N angles 79.68(7)°–98.93(7)° indicate a slightly distorted coordination geometry for Cu(II). Two neighboring Cu(II) ions related to an inversion center are bridged by two EO-N_3_ bridges (N1 and N1A) resulting in a dinuclear [Cu_2_(EO-N_3_)_2_] unit, in which the Cu–N–Cu bridging angle and Cu···Cu distance are 100.32(8)° and 3.093(1) Å, respectively (Figure 3a). Each Cu_2_ unit is further linked to four identical neighboring Cu_2_ units by four *μ*-*N*,*N*-pyrimidyl groups from four bpym ligands, resulting in a 2D subnet that is orientated parallel to the *bc* crystal plane (Figure 3b), where the Cu···Cu distance spanned by the *μ*-pyrimidyl group is 6.204(7) Å. In the layer, the Cu_2_ moiety systematically alternates in two different orientations with the dihedral angle of the Cu_2_N_2_ planes being 63.02(5)°. As shown in Figure 3c, the bpym ligands in **2** acts a twisted *μ*_4_-*N*,*N*′,*N*′′,*N*′′′-tetratopic connector (Scheme 1b) with the dihedral angle of the two pyrimidyl rings of 46.7(3)°, and extends Cu(II)-pyrimidine subnets alternately to a 3D framework along the *a* axis in a cavity-above-cavity (···AAAA···) fashion. The nearest interlayer Cu···Cu distance is 8.53 Å. Indeed, due to the twisting of two pyrimidyl rings of the bpym ligands, the Cu(II) centers are displaced from the mean plane of the *μ*-pyrimidyl rings (ca. 0.179 and 0.423 Å).

**Figure 4:**
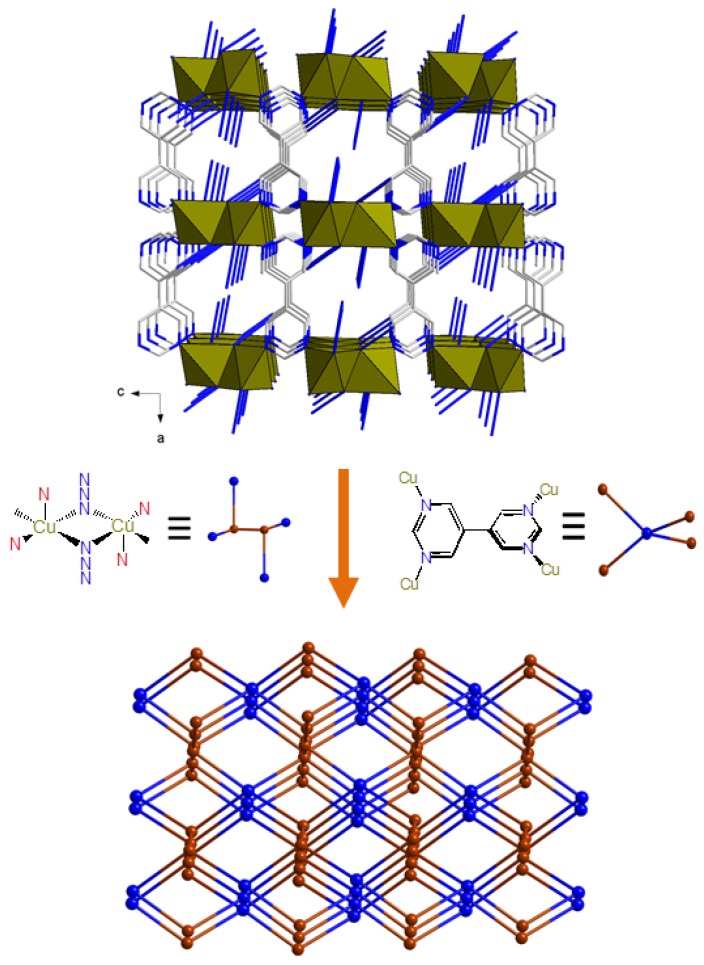
Schematic representation of the (3,4)-connected **tfi** topologic network Compound **2**; the EO- and EE-azides are represented by green; the bpym and Cu center are represented by blue and brown spheres, respectively.

From a topology point of view of the complicated 3D framework of **2**, each square pyramidal Cu(II) center connecting to three neighboring Cu(II) centers by one basal *μ*-pyrimidyl ring, one apical *μ*-pyrimidyl ring and a pair of basal EO-N_3_ group can be regarded as a pyramidal three-connected node, while each twisted *μ*_4_-bpym ligand can be considered to be an elongated tetrahedral four-connected node. A TOPOS analysis [28] indicates that the net of **2** can be rationalized as a binodal (3,4)-connected **tfi** topology with the Schläfli symbol (6^2^.8^4^)(6^2^.8)_2_ as illustrated in Figure 4.

### 3.3. Magnetic Properties

**Figure 5:**
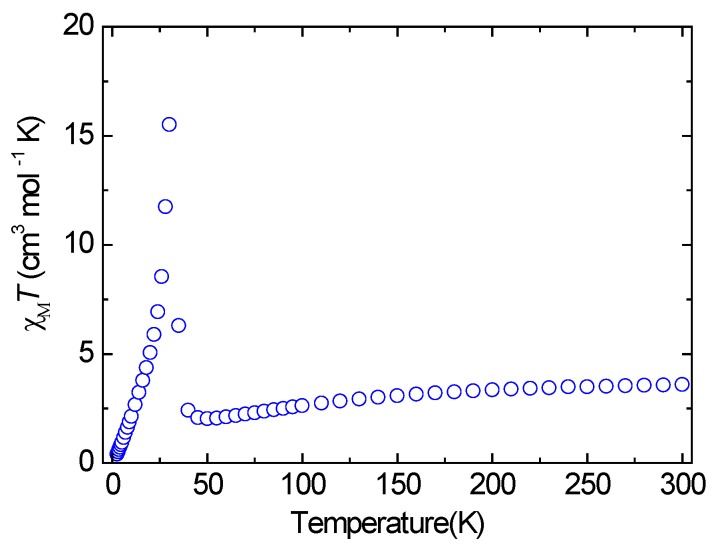
Plot of χ_M_*T* vs. *T* of Compound **1** in an applied field of 1 kOe from 2–300 K.

The variable temperature dependence of magnetic susceptibility was performed on powdered samples of Compounds **1** and **2** under 1000 Oe in the temperature range of 2–300 K. The temperature dependence of the χ_M_*T* and χ_M_ plots of Compound **1** are shown in Figure 5 and Figure S3, respectively. As shown in Figure 5, the χ_M_*T* value per Co(II) of **1** at 300 K is 3.61 cm^3^ mol^−1^ K, which is larger than the expected value for a high-spin Co(II) ion (1.87 cm^3^ mol^−1^ K with *g* = 2.0). With decreasing temperature, the χ_M_*T* decreases gradually, reaching a minimum value of 2.39 cm^3^ mol^−1^ K at 50 K, then rises rapidly to reach a sharp maximum value of 15.52 cm^3^ mol^−1^ K at 30 K and finally drops again to 0.41 cm^3^ mol^−1^ K at 2.0 K. The data above 100 K obey the Curie–Weiss law with a Curie constant *C* = 4.40 cm^3^ mol^−1^ K and a Weiss constant θ = −64.7 K (Figure S3). The large negative value of θ and the initial decrease of χ_M_*T* above 50 K could be the concurrent operation of the spin-orbit coupling effect of the octahedral Co(II) ions, ligand field effects and antiferromagnetic coupling between the adjacent Co ions. The abrupt increase in χ_M_ (Figure S4) and χ_M_*T* below 50 K is indicative of the spontaneous, ferromagnetic-like magnetization, due to spin canting. The final rapid drop in χ_M_*T* and χ_M_ at temperatures below 30 K can be attributed to saturation effects and/or a further antiferromagnetic interaction. Generally speaking, the spin-canted behavior can arise from two contributions: (1) the occurrence of an antisymmetric exchange, the so-called Dzyaloshinskii−Moriya interaction; and (2) the presence of single-ion magnetic anisotropy. The existence of an inversion center between adjacent spin centers could result in the disappearance of the antisymmetric exchange. Taking into account the structural features of Compound **1**, although the adjacent spin Co(II) ions are related to a crystallographic inversion center, the Co(II) ions are connected by 2_1_ helices with opposite chiralities through EE-N_3_ groups. The different orientations of opposite chirality could bring the existence of antisymmetric exchange, thus resulting in spin-canted behavior. Moreover, the distortion of the octahedral Co(II) site might display considerable anisotropy, which also contributes to the spin canting in **1**. Some similar results have been reported for other Co(II) compounds [29,30,31,32].

**Figure 6:**
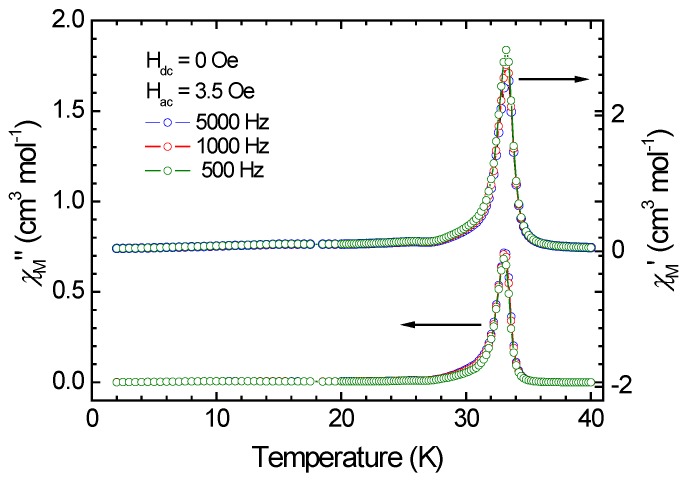
In-phase (χ′) and out-of-phase (χ′′) of the ac magnetic susceptibilities in a zero applied dc field and a 3.5-G ac field at the indicated frequencies for Compound **1**.

To investigate low-temperature magnetic behavior, zero-field-cooled (ZFC) and field-cooled (FC) magnetizations were performed for **1** at 10 Oe in the range of 2.0–40 K (Figure S5). Upon cooling, both ZFC and FC magnetization show an abrupt increase below 35 K and a divergence below 33.0 K, suggesting the occurrence of the formation of an ordered state and the existence of an uncompensated moment below the critical temperature (*T_c_*) of 33.0 K. Indeed, the change in FC magnetizations at 31.0 and 18.0 K can be attributed to spin reorientation. The obtained results from the ZFC/FC magnetizations are compatible with measurements of alternating current (ac) magnetic susceptibility at different frequencies under *H*_dc_ = 0 Oe and *H*_ac_ = 3.5 Oe (Figure 6). As can be seen in Figure 6, both in-phase (χ_M_′) and out-of-phase (χ_M_′′) signals exhibit maxima at ca. 33.0 K, and this maximum position is frequency-independent. This confirms the occurrence of magnetic ordering that is ferromagnetic-like due to spin canting, which is consistent with the ZFC/FC magnetization data. The observance of non-zero χ_M_′′ signals below *T*_c_ is due to an uncompensated moment, and consequently, coercive behavior would be expected. It should be noted that the χ_M_′ and χ_M_′′ peaks are unsymmetrical in shape, with the low-temperature side showing some frequency dependence. This indicates the presence of a dynamic relaxation process [33].

**Figure 7:**
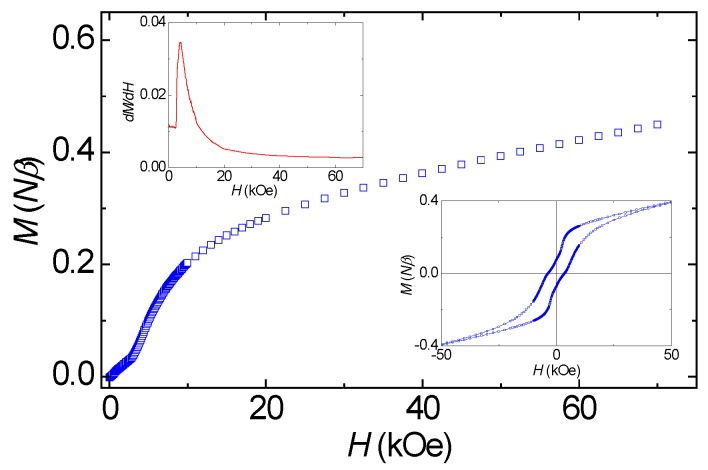
Field dependence of the magnetization of **2** at 2.0 K. The insets give the derivation of *M* vs. *H* and a blow-up of the hysteresis loop.

To further study the magnetic properties of **1**, the isothermal field-dependent magnetization of **1** was measured below *T*_C_ (Figure 7). At 2.0 K, the magnetization shows an abrupt increase at a field above 3.2 kOe with a sigmoidal shape, then reaching a value of 0.45 *Nβ* at 70 kOe. Such sigmoidal magnetization clearly points out a field-induced magnetic transition in the metamagnetism. In a metamagnetic transition, the net moments that are aligned antiparallel by coupling of weak antiferromagnetic interactions are overcome by an external field. Thus, the slow increase in magnetization in the low field region (0–3.2 kOe) indicates the presence of antiferromagnetic interactions of the spin-canted layers, and the rapid rise in the 3.2–11.2 kOe region indicates that the interlayer antiferromagnetic interactions are overcome by the applied field. Indeed, the critical field of transition, *H*_C_, at 2.0 K was estimated to be about 4.4 kOe, as determined by *dM*/*dH* (Figure 7, inset). The *M* value of 0.45 *Nβ* at 70 kOe is far below the expected value of saturation of 2–3 *Nβ* for an isotropic high-spin Co(II) system, confirming the antiferromagnetic nature of Compound **1**. Such field-induced magnetic transition of metamagnetism is frequently observed in layer or chain systems with strong anisotropy and competing interactions [34,35,36,37]. The hysteresis loop is obvious at 2.0 K (Figure 7, inset), which shows a remnant magnetization of 0.074 *Nβ* and a coercive field of 3.5 kOe, indicating that **1** has a hard magnet behavior. Based on the remnant magnetization at 2.0 K, the canting angle is estimated to be approximately 2.0°. It is noteworthy that the hysteresis loop of Compound **1** at 2.0 K exhibits several reproducible steps when the field is swept from one end to the other end of the loop, which is characteristic of a system with uniaxial symmetry [38]. 

**Figure 8:**
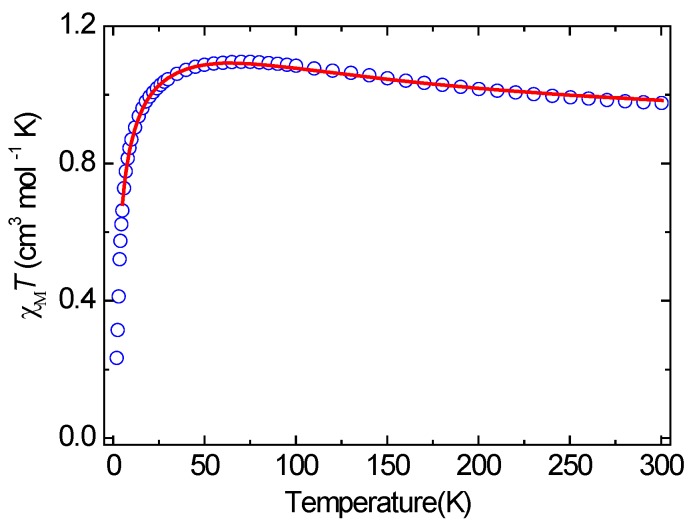
Plot of χ_M_*T* vs. *T* of Compound **2** in an applied field of 1 kOe from 2–300 K. The solid line represents the best fit.

The temperature-dependent χ_M_*T* for Compound **2** in the range of 2.0–300 K under a 1.0 kOe of applied field is shown in Figure 8. The χ_M_*T* value at 300 K is 0.98 cm^3^ mol^−1^ K, which is higher than the expected value of 0.75 cm^3^ mol^−1^ K for an isolated Cu(II) ion (*S* = ^1^/_2_ and *g* = 2.0). The χ_M_*T* value increases gradually with the decrease of temperature, reaching a smooth maximum of 1.10 cm^3^ mol^−1^ K at 90 K, indicating the existence of ferromagnetic interactions. The χ_M_ data above 100 K obey the Curie–Weiss law with a *C* = 0.93 cm^3^ mol^−1^ K and a θ = 15.79 K (Figure S6). The positive value of θ confirms that ferromagnetic coupling is dominant. Upon further cooling, the χ_M_*T* value abruptly decreases to 0.23 cm^3^ mol^−1^ K at 2.0 K, implying overall antiferromagnetism in the low-temperature range. The Néel temperature (*T*_N_), as deduced from the maxima value of *d*χ_M_/*dT*, was *T*_N_ = 3.05 K. Considering the crystal structure, the analysis of the magnetic properties of **2** was carried out by using the structure as a square lattice of dimeric units (layer of dimmers). The expression of the magnetic susceptibility for the dinuclear moiety (χ_dimer_) was deduced by an isotropic Heisenberg Hamiltonian: *H* = −*JS*_Cu1_*S*_Cu2_, where *J* is the magnetic interaction through the double EO-N_3_ bridges. The dinuclear units were presumed to have an effective spin that was associated with the following Equation (1):

(1) $$S_{\mathrm{eff}}(S_{\mathrm{eff}}+1)=\frac{2k\chi_{\mathrm{dimer}}T}{\left(Ng^{2}\beta^{2}\right)}$$

The expression of Equation (2) deduced by the Curely classical spins for a square lattice was then used to define the magnetic properties of the “layer of dimers” of Compound **2**.

(2) $$\chi_{M}=\left[\frac{Ng^{2}\beta^{2}S_{\mathrm{eff}}(S_{\mathrm{eff}}+1)}{3kT}\right]\left[\frac{{\left(1+u\right)}^{2}]}{{\left(1-u\right)}^{2}}\right]$$

where *u* is the Langevin function, *u* = conth[*J*_eff_′*S*_eff_(*S*_eff_ + 1)/*kT*] − *kT*/[*J*_eff_′*S*_eff_(*S*_eff_ + 1)] with *J*_eff_′ accounting for the effective magnetic interactions between the Cu_2_ units through the *μ*-*N*,*N*′-pyrimidyl bridges. The best fit above 40 K leads to values of *g* = 2.17, *J* = 62.2 cm^−1^, and *J*_eff_′ = −1.90 cm^−1^. The *J* value from the fitting result is in good agreement with those for related [Cu_2_(EO-N_3_)_2_] compounds and confirms that the magnetic interaction mediated by double EO-N_3_ is ferromagnetic [36].

**Figure 9:**
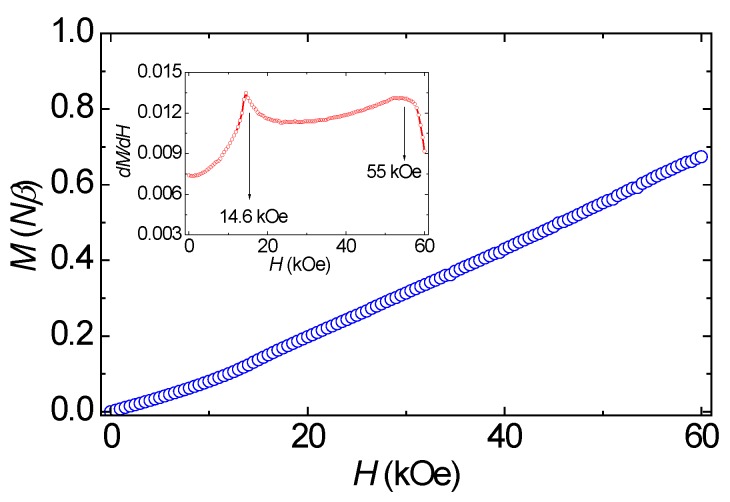
The isothermal magnetization of **2** at 2.0 K. The inset gives the derivation of *M* vs. *H*.

To study the low temperature magnetic behavior of Compound **2**, zero ZFC/FC magnetizations down to 2.0 K were collected (Figure S7). The ZFC/FC magnetizations show a maximum at 3.1 K with the increase of temperature without any divergence indicating the existence of antiferromagnetic ordering below 3.1 K, which is consistent with the dc magnetic data. Antiferromagnetic ordering was confirmed by the ac magnetic susceptibility measurements, in which the χ′ signals increases with decreasing temperature and reach a maximum at 3.1 K without the presence of the χ″ signal (Figure S8). Isothermal magnetizations *M*(*H*) of **2** were performed at 2.0 K up to ±60 kOe (Figure 8), in which no hysteresis loop was observed. However, magnetization vs. field findings show interesting behavior; in the low field region (*H* < 15 kOe), the magnetization increases slowly, and after a steady increase, it turns linear until the field is about 60 kOe, where a second change, although a pronounced loss, is observed. Such magnetization behavior is an indication of a file-induced spin-flop (SF) magnetic transition, as has been reported for other antiferromagnets with a small degree of single-ion and/or exchange anisotropy [39]. For an SF transition, the antiparallel aligned spin “flops” to perpendicularity with the applied magnetic field. The spin-flop field (*H*_SF_) and critical field (*H*_C_) for **2** are found at ca. 14.6 and 55.0 kOe, respectively, as deduced from the peak position of *dM*/*dH* (Figure 9, inset). The *M* value of 0.67 *Nβ* at 60 kOe is less than the expected value of 1.0 *Nβ* for saturation that would be predicted for a Cu(II) ion (*S* = 1/2, *g* = 2.0). To further substantiate the existence of spin-flop, the field dependence of the ac magnetic susceptibilities, χ_M_′(*H*), at 2.0 K was collected (Figure S8). The χ_M_′(*H*) shows a sharp peak and a round maximum for the *H*_SF_ and *H*_C_ transitions, respectively, which is consistent with the results of *dM*/*dH*.

Indeed, solid state X-band EPR spectra for Compounds **1** and **2** were collected at 77 K (Figure S9). For Compound **1**, the spectra exhibit a broad and asymmetric band, where the peak-to-peak ΔHp-p is 1250 G, and the center is at 1800 G. The hyperfine structure of the 100% abundant ^59^Co isotope (^59^I = ^7^/_2_) is not observed. This result suggests a large anisotropic g-factor and the magnitude of the exchange coupling between each Co(II) ions, which are larger than the magnitude of the hyperfine coupling parameter A. Thus, the hyperfine structure collapses and results in a single collapsed peak. In contrast, the spectra of Compound **2** show only one broad signal at 77 K. This broadening signal in the spectrum might be due to spin relaxation.

## 4. Conclusions

The introduction of a semi-flexible *N*-donor ligand, 5,5′-bipyrimidin (bpym), permitted two new Co(II)- and Cu(II)-azido coordination polymers to be synthesized. Both **1** and **2** are 3D frameworks with pillared-layer architectures. The structure of Compound **1** is composed of Co(II)-azido layers, formed from [Co_2_(EO-N_3_)_2_] motifs, single end-to-end (EE) azido bridges and linear *μ*_2_-bpym pillars with a **BN** topology. In Compound **2**, the bpym ligand shows a twisted *μ*_4_-bridging mode, which not only connects the adjacent [Cu_2_(EO-N_3_)_2_] units to a layer, but also acts as a pillar to connect the layers to form a 3D structure with the **tfi** topology. Magnetic investigations reveal that Compound **1** exhibits ferromagnetic-like behavior and magnetic ordering due to spin canting with *T*_C_ = 33.0 K and shows the field-induced magnetic transitions of metamagnetism below *T*_C_. Compound **2** is an antiferromagnet with magnetic ordering of *T*_N_ = 3.05 K and furthers the field-induced magnetic transition of spin-flop below the *T*_N_. The studies show that the use of a semi-flexible ligand such as a 5,5′-bipyrimidin derivative is particularly promising for synthesis in the construction of polymeric networks of CPs with versatile topological structures and bulk magnetic behavior.

## Figures and Tables

**Table 1:** Crystallographic data for **1** and **2**.

Compound	1	2
Formula	C_8_H_6_CoN_10_	C_4_H_8_CuN_8_
Fw	301.16	226.69
Crystal system	Monoclinic	Monoclinic
Space group	*P*2_1_/c	*C*2/c
*a*/Å	9.9130(7)	16.0377(18)
*b*/Å	8.1550(6)	6.1184(7)
*c*/Å	13.1987(10)	14.2463(16)
α/°	90	90
β/°	90.1186(14)	92.031(2)
γ/°	90	90
*V*/Å^3^	1066.99(14)	1397.0(3)
Z	4	3
*T*/K	150(2)	150(2)
*D*_c_/g cm^−3^	1.875	2.156
μ/mm^−1^	1.613	3.084
(Δρ)max, min/e Å^−3^	0.559, −0.572	0.429, −0.413
Measured/independent (*R*int) reflections	9357/2454(0.0326)	4917/1668(0.0213)
Observed reflections (*I* *>* 2σ(*I*))	9357	4917
Goodness-of-fits on *F*^2^	1.225	1.063
*R*_1_^1^, w*R*_2_^2^ (all data)	0.0445, 0.0831	0.0273, 0.0577
*R*_1_^1^, w*R*_2_^2^ (*I* > 2σ(I))	0.0403, 0.0814	0.0230, 0.0554

^1^ *R*_1_ (Σ||*F*_O_| − |*F*_C_||)/Σ|*F*_O_|; ^2^ w*R*_2_ = [Σw|*F*_O_^2^ − *F*_C_^2^|^2^/Σw(*F*_O_^4^)]^1/2^.

**Table 2:** Selected bond distances (Å) and angles (°) for Compounds **1** and **2**.

**1** ^a^			
Co-N(1)	2.161(2)	Co-N(3) ^3^	2.163(2)
Co-N(5)	2.147(2)	Co-N(8)	2.113(2)
Co-N(5) ^2^	2.158(2)	Co-N(10) ^1^	2.119(2)
N(8)-Co-N(10) ^1^	97.50(9)	N(5)-Co-N(1)	90.58(8)
N(8)-Co-N(5)	92.98(8)	N(5) ^2^-Co-N(1)	92.07(8)
N(10) ^1^-Co-N(5)	169.21(8)	N(8)-Co-N(3) ^3^	87.73(9)
N(8)-Co-N(5) ^2^	171.71(8)	N(10) ^1^-Co-N(3) ^3^	91.62(8)
N(10)^1^-Co-N(5) ^2^	90.79(8)	N(5)-Co-N(3) ^3^	91.37(8)
N(5)-Co-N(5) ^2^	78.73(8)	N(5) ^2^-Co-N(3) ^3^	92.50(8)
N(8)-Co-N(1)	87.91(9)	N(1)-Co-N(3) ^3^	175.31(8)
N(10) ^1^-Co-N(1)	87.24(8)	N(6)-N(5)-Co ^2^	123.57(17)
Co-N(5)-Co ^2^	101.27(8)		
**2** ^b^			
Cu(1)-N(4)	1.9661(18)	Cu(1)-N(1) ^4^	2.0312(16)
Cu(1)-N(1)	1.9969(16)	Cu(1)-N(7)	2.0420(16)
N(1)-Cu(1) ^4^	2.0312(16)		
N(4)-Cu(1)-N(1)	98.93(7)	N(4)-Cu(1)-N(7)	90.05(7)
N(4)-Cu(1)-N(1) ^4^	170.55(8)	N(1)-Cu(1)-N(7)	169.75(7)
N(1)-Cu(1)-N(1) ^4^	79.68(7)	N(1) ^4^-Cu(1)-N(7)	92.26(6)
N(2)-N(1)-Cu(1)	123.71(14)	N(5)-N(4)-Cu(1)	124.42(15)
N(2)-N(1)-Cu(1) ^4^	120.02(13)	C(3)-N(7)-Cu(1)	117.55(12)
Cu(1)-N(1)-Cu(1) ^4^	100.32(7)	C(4)-N(7)-Cu(1)	125.44(13)

^a^ Symmetry transformations used to generate equivalent atoms: ^1^ −x + 1, y − 1/2, −z + 1/2; ^2^ −x + 1, −y, −z; ^3^ x − 1, y, z; ^b^ symmetry transformations used to generate equivalent atoms: ^4^ −x + 1/2, −y + 3/2, −z + 1.

## Supplementary Materials

The following are available online at http://www.mdpi.com/2073-4360/10/3/229/s1, Figure S1: Simulated PXRD pattern (blue) and experimental PXRD pattern (red) of Compounds (a) **1** and (b) **2**. Figure S2: Thermogravimetric (TG) analysis diagrams of Compounds **1** (red line) and **2** (blue line). Figure S3: Plot of χ_M_^−1^ (○) vs. *T* of Compound 1. The solid line represents the best fit χ_M_^−1^ above 100 K with a Curie–Weiss law. Figure S4: Plot of χ_M_ (○) vs. *T* of Compound **1**. Figure S5: ZFC/FC magnetizations of Compound **1** at a field of 10 Oe. Figure S6: Plot of χ_M_^−1^ (○) vs. T of Compound **2**. The solid line represents the best fit χ_M_^−1^ above 100 K with a Curie–Weiss law. Figure S7: In-phase (χ′) and out-of-phase (χ′′) of the ac magnetic susceptibilities in a zero applied dc field and a 3.5 G ac field at the indicated frequencies for Compound **2**. Figure S8: χ_M_′ vs. *H* plots for field dependence of the ac magnetic susceptibilities in a zero applied dc field and in a 3.5 Oe ac field and 100 Hz at 2.0 K for **2**. Figure S9: EPR spectra of (a) Compound **1** and (b) Compound **2**.
