# Supplementary material for: Two New Three-Dimensional Pillared-Layer Co(II) and Cu(II) Frameworks Involving a [M2(EO-N3)2] Motif from a Semi-Flexible N-Donor Ligand, 5,5′-Bipyrimidin: Syntheses, Structures and Magnetic Properties
Source: Polymers (Basel). 2018 Feb 26;10(3):229. doi: 10.3390/polym10030229 (PMC6415140; doi:10.3390/polym10030229)
Supplement: Supplementary file 1 [file polymers-10-00229-s001.pdf]

(**a**)

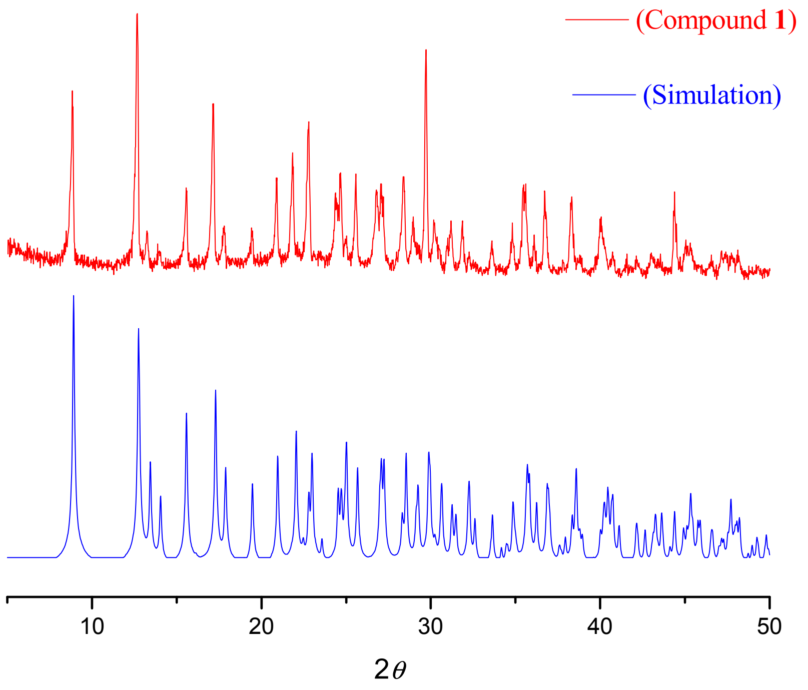

(**b**)

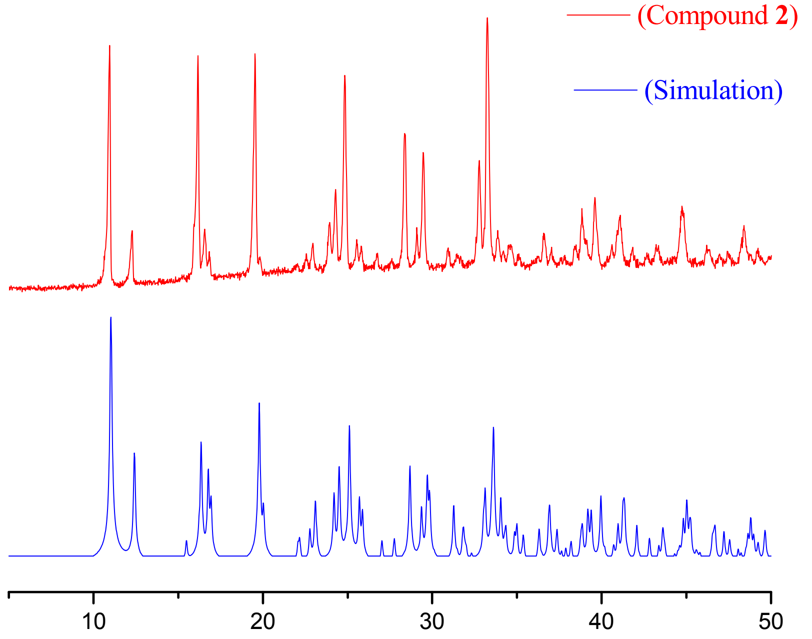

Figure S1. Simulated PXRD pattern (blue) and experimental PXRD pattern (red) of compounds **(a) 1** and (**b**) **2.**

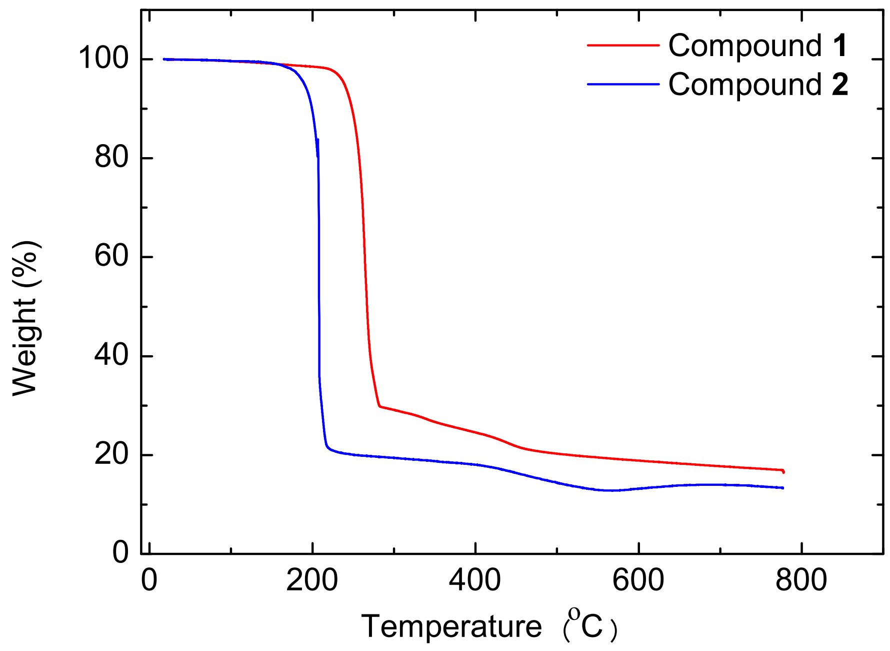

Figure S2. Thermogravimetric (TG) analysis diagrams of compounds **1** (red line) and **2** (blue line).

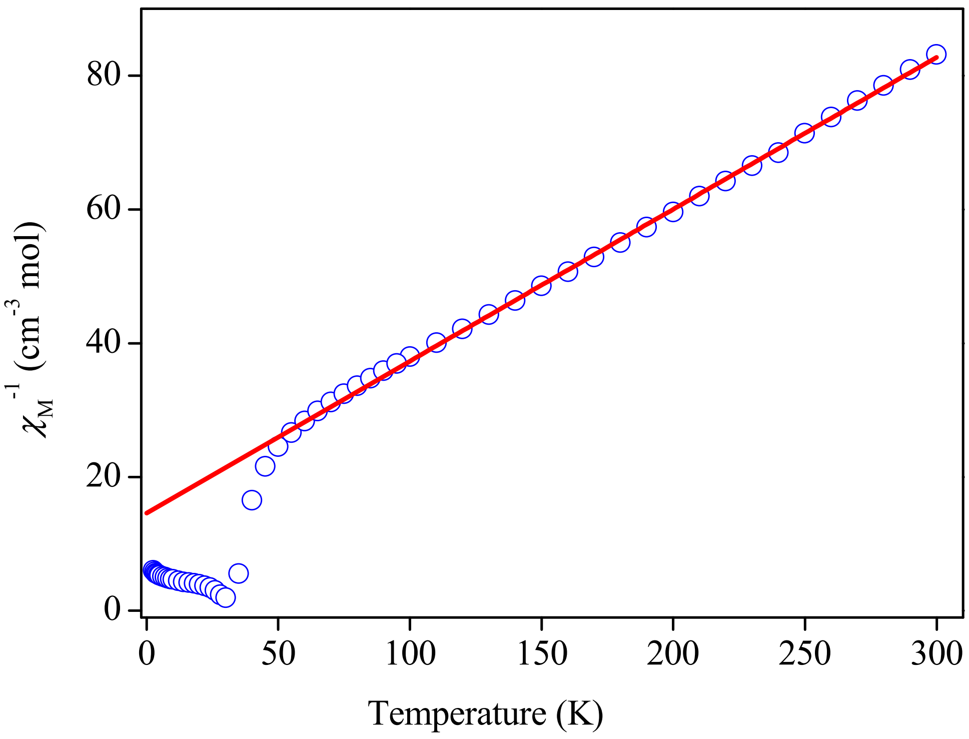

Figure S3. Plot of χ_M^{-1} (○) vs. T of compound **1**. The solid line represents the best fit χ_M^{-1} above 100 K with a Curie–Weiss law.

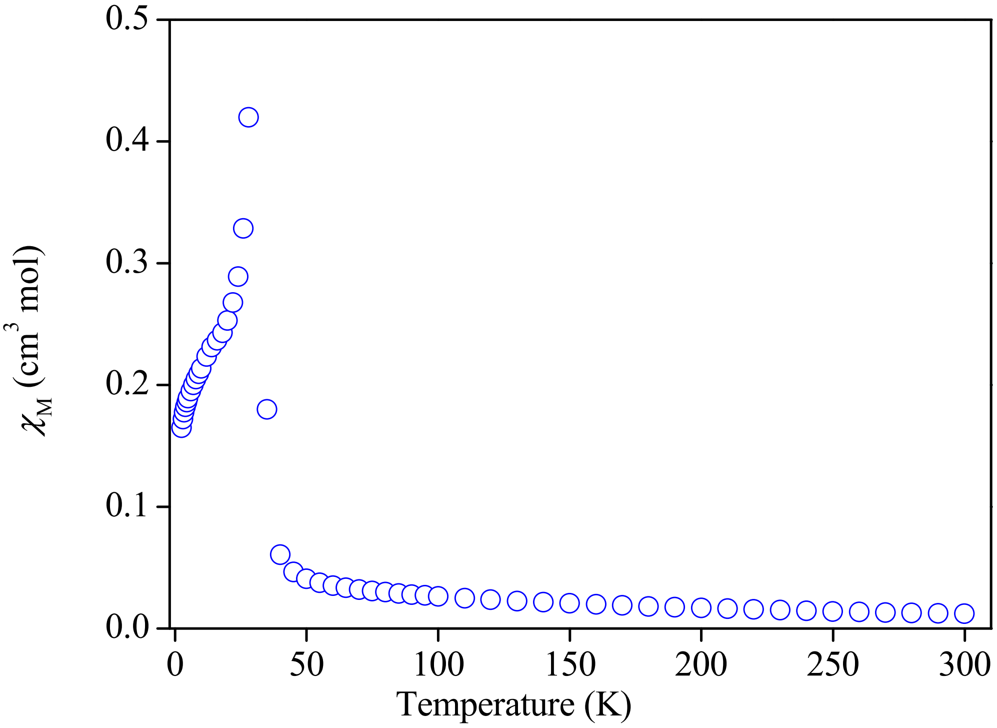

Figure S4. Plot of χ_M (○) vs. *T* of compound **1**.

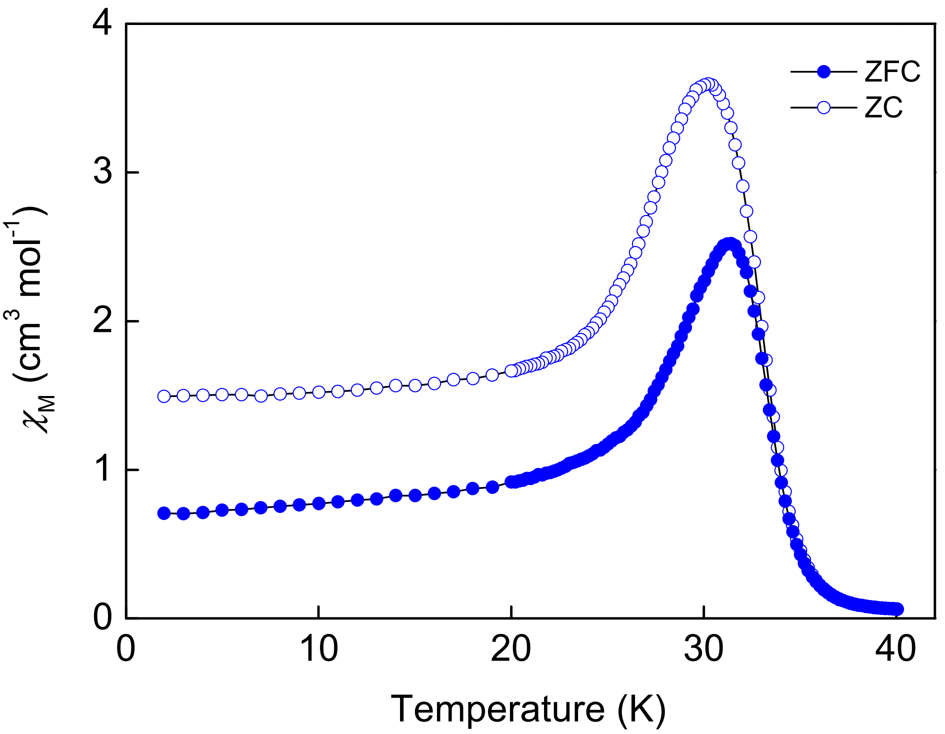

Figure S5. ZFC/FC magnetizations of compound **1** at the field of 20 Oe.

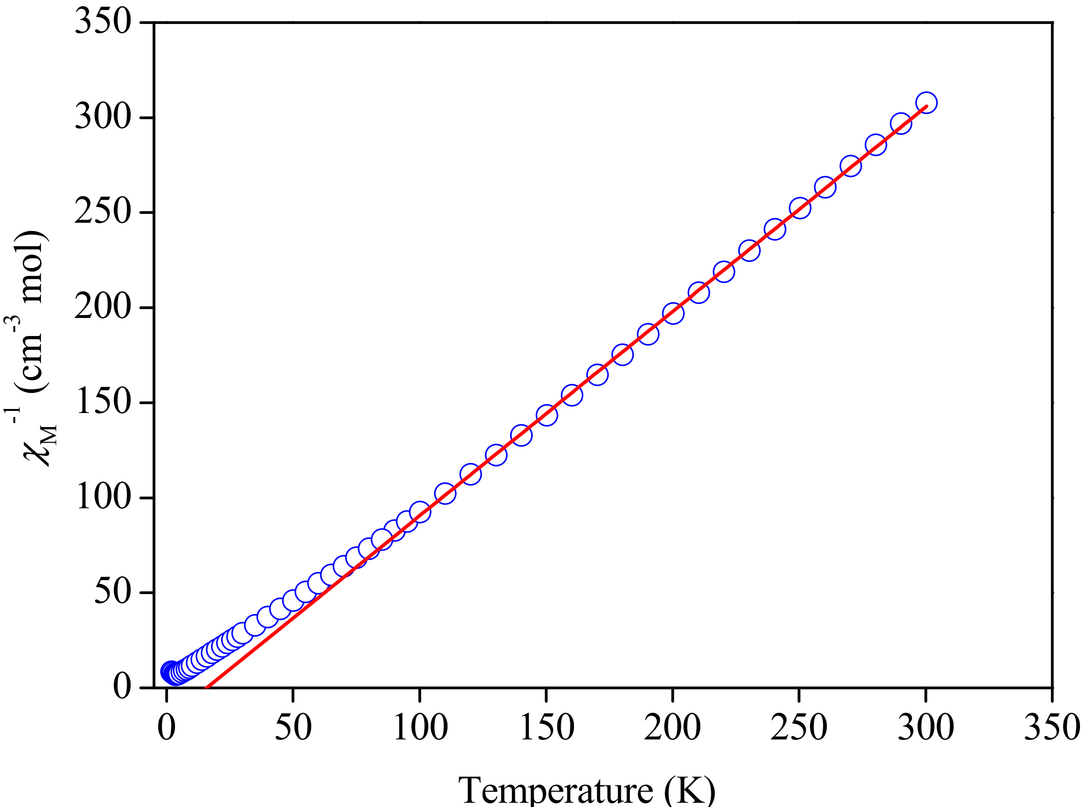

Figure S6. Plot of χ_M^{-1} (○) vs. T of compound **2**. The solid line represents the best fit χ_M^{-1} above 90 K with a Curie–Weiss law.

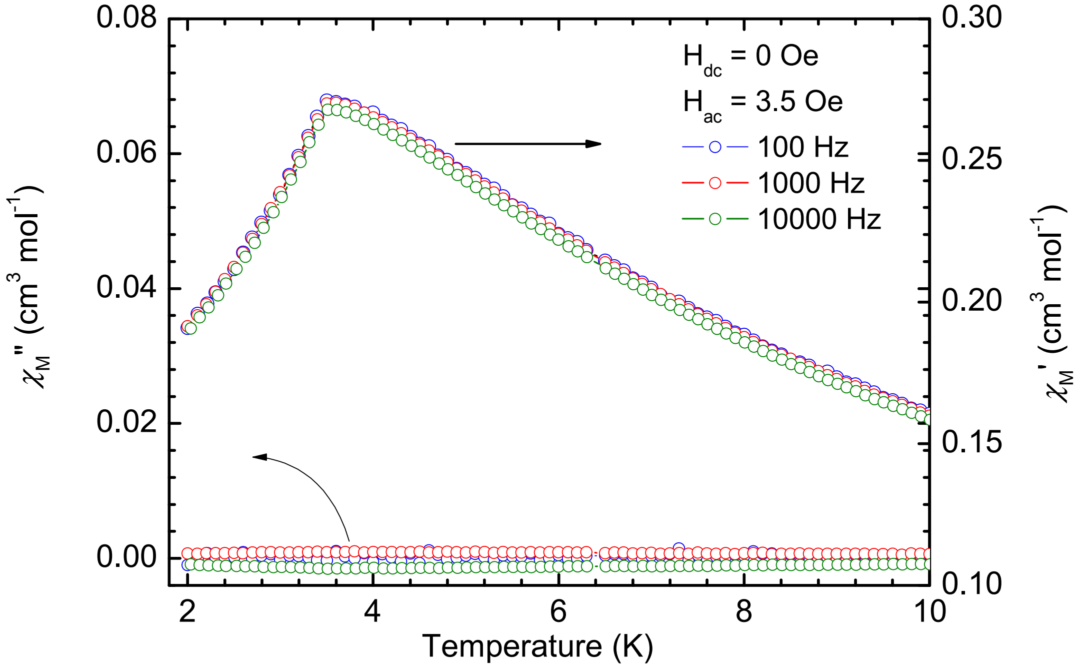

Figure S7. In phase (χ′) and out-off phase (χ″) of the ac magnetic susceptibilities in a zero applied dc field and a 3.5 G ac field at the indicated frequencies for compound **2**.

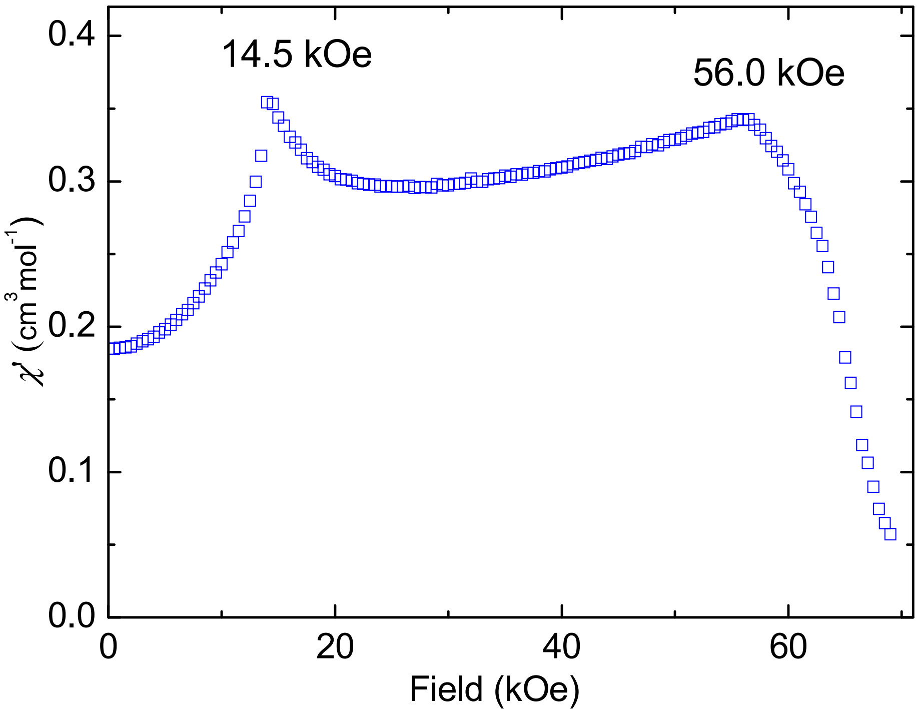

Figure S8. χ_M' vs. *H* plots for field dependence of the ac magnetic susceptibilities in a zero applied dc field and in a 3.5 Oe ac field and 100 Hz at 2.0 K for **2**.

(**a**)

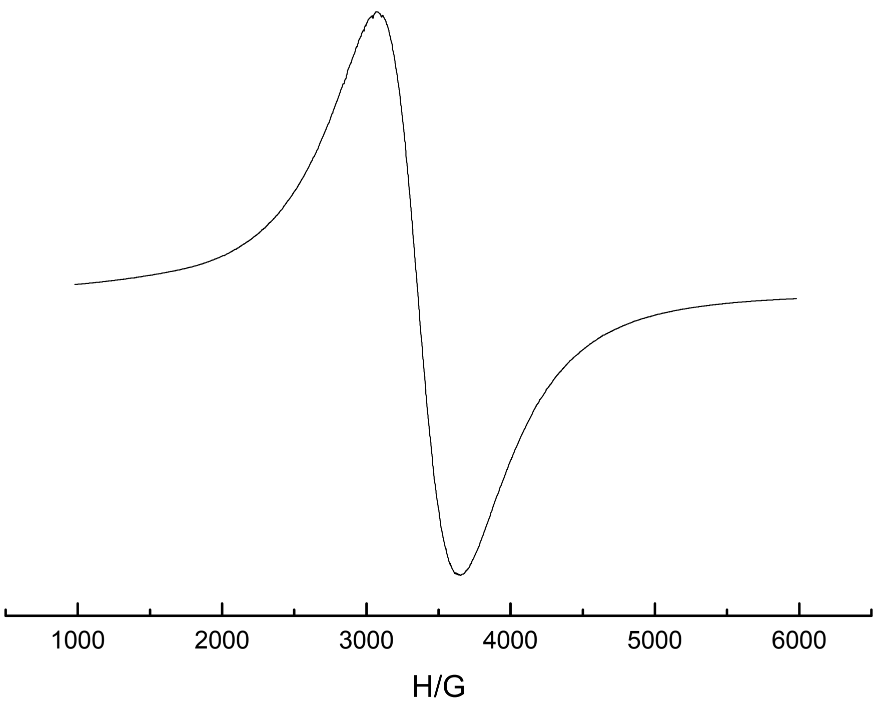

(**b**)

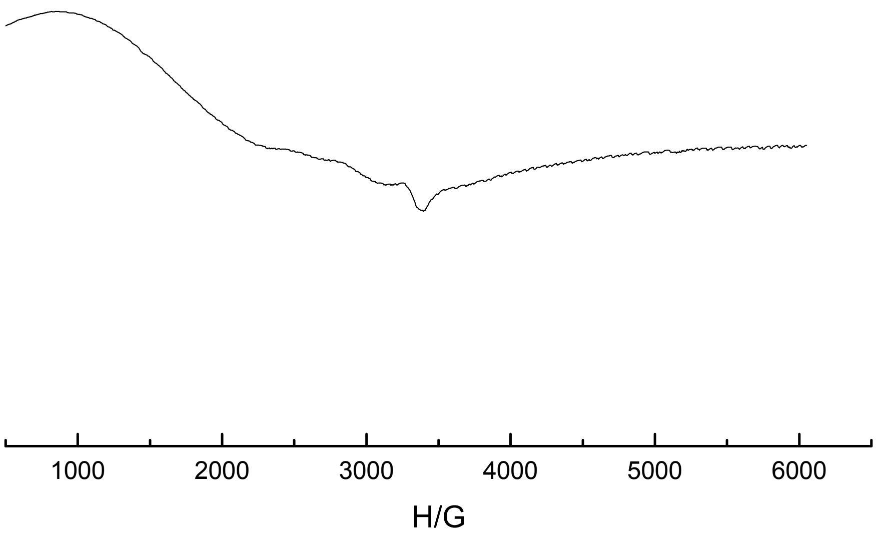

Figure S9. EPR spectra of (**a**) compound **1** and (**b**) compound **2**.
